# Age and time trends of dairy intake among children and adolescents of the DONALD study

**DOI:** 10.1007/s00394-021-02555-7

**Published:** 2021-04-21

**Authors:** Eva Hohoff, Ines Perrar, Nicole Jancovic, Ute Alexy

**Affiliations:** Department of Nutritional and Food Sciences, Nutritional Epidemiology, University of Bonn, DONALD Study Dortmund, Heinstück 11, 44225 Dortmund, Germany

**Keywords:** Dairy, Dairy intake, Time trends, Age trends, Children, Adolescents

## Abstract

**Purpose:**

To describe age and time trends in dietary intake of Total Dairy (TD) (g/1000 kcal Total Energy Intake) and types of dairy (weight percent of total dairy intake, w%TD) represented as Low Fat Dairy (LFD), High Sugar Dairy (HSD), Fermented Dairy (FD) and Liquid Dairy (LD) among children and adolescents in Germany.

**Methods:**

Overall, 10,333 3-day dietary records kept between 1985 and 2019 by 1275 DONALD participants (3.5–18.5 years; boys: 50.8%) were analyzed using polynomial mixed-effects regression models.

**Results:**

TD intake decreased with age (♂: linear trend *p* < 0.0001; ♀: linear and quadratic trend *p* < 0.0001), whereas FD (♀: linear, quadratic, cubic trend *p* ≤ 0.02) increased slightly in girls. HSD (♂: linear, quadratic, cubic trend *p* ≤ 0.004; ♀: linear, quadratic, cubic trend *p* ≤ 0.005) and LD (linear, quadratic trend *p* ≤ 0.0002) decreased with age. In terms of time trends, TD intake decreased in the last three decades, especially since 1995 (quadratic trend for ♂ 0.0007 and ♀ *p* = 0.004). LFD intake increased until 2010 and decreased thereafter (linear, quadratic, cubic trend *p* < 0.0001). HSD decreased until 1995, then increased until 2010 and decreased again afterwards (♂: linear, quadratic, cubic trend *p* ≤ 0.001; ♀: linear, quadratic, cubic trend *p* ≤ 0.003). While FD intake increased linear (in both ♂ and ♀: *p* < 0.0001), LD intake decreased (linear, quadratic trend *p* ≤ 0.03).

**Conclusion:**

Our results showed changes in dairy consumption patterns among children and adolescents over the past three decades, demonstrating a decrease in TD intake with age and time, and a shift from liquid to solid dairy products with a simultaneous increase in fermented dairy products, while LFD and HSD fluctuated over time. Further evaluations will examine the health significance of these consumption patterns.

**Supplementary Information:**

The online version contains supplementary material available at 10.1007/s00394-021-02555-7.

## Introduction

In recent years, several meta-analyses examined associations between dairy consumption and health outcomes. A current meta-analysis of prospective cohort studies in children indicated an inverse relationship between total dairy intake and body fat ratio as well as the risk of overweight/obesity [1]. Further meta-analyses of intervention trials and observational studies found a positive association of dairy related to bone mineral content in children [2] and an inverse relationship between dairy intake and diabetes mellitus 2 [3].

However, dairy is a heterogeneous food group in terms of nutrient content and differs mainly in fat and added sugar content. The relevance of nutrient contents was shown in three meta-analyses of observational studies showing an inverse association between the amount of dairy consumption and diabetes type 2 risk only for fat reduced dairy products [4–6]. Since dairy products contribute to a large extent (12%) to the intake of free sugar among children and adolescents [7], sugar content of dairy should also be of interest.

Furthermore, processing methods may enhance interactions between nutrients in dairy, which may modify the metabolic effects of dairy consumption [8]. A number of studies have attributed a protective effect against various diseases such as diabetes type 2 to fermented dairy [4, 9]. In the European Prospective Investigation into Cancer and Nutrition (EPIC) study as well as in the National Health and Nutrition Examination Survey (NHANES) study, the consumption of yogurt was also associated with a reduced weight gain [9, 10]. Furthermore, the consistency of dairy products, i.e., liquid or solid, may also be relevant, since liquid foods are discussed to have a less satiation impact than solid foods [11, 12].

Since dairy intake in childhood was associated with healthy eating patterns and lifestyle factors in adults [13], age and time trends in dairy intake as well as in the intake of different types of dairy among children and adolescence are of particular interest. In a previous dairy trend analysis with healthy subjects of the German DONALD (DOrtmund Nutritional and Anthropometric Longitudinally Designed) study from 1986 to 2001 total consumption of dairy products remained at least stable over 4 years, but a shift from dairy beverages towards dairy foods was observed. In children and adolescents, dietary habits change with age, e.g., during puberty [14]. This is important considering the fact that puberty may be a critical period for the development of overweight [15].

However, in the last 20 years, new products and increasingly dairy alternatives were introduced. Therefore, the aim of the present analyses was to examine current age and time trends in the intake of dairy and dairy types among 3.5- to 18.5-year-old DONALD participants over three decades from 1985 to 2019.

## Methods

### Study sample

The DONALD study is an ongoing open cohort study collecting information on nutrition, growth, development and metabolism of children and adolescents in Dortmund, Germany. Since 1985, the study has enrolled approximately 35–40 healthy infants each year. Annual examinations include 3-day weighing protocols, anthropometric measurements, medical examinations, lifestyle interviews, and 24-h urine samples. Parental examinations occur every 4 years. Further details of the study are described elsewhere [16, 17].

The study is approved by the ethics committee of the University of Bonn according to the guidelines of the Declaration of Helsinki. At the beginning of the present analysis (June 2019), a total of 17,782 records was available. Records were excluded if they were incomplete (< 3 days, *n* = 192) or if they were from < 3.5-year-olds (*n* = 20,700) or > 18.5-year-olds (*n* = 1491). Records with intake of breast milk or formula were also eliminated (*n* = 156). Hence, for the present evaluation, we analyzed 10,333 complete dietary records from 1275 DONALD study participants (*n* = 648 boys, 627 girls).

### Dietary assessment

In the DONALD study, dietary intake is recorded on the basis of 3-day weighed dietary records. The food and beverages consumed, as well as leftovers, are weighed and recorded by parents or by the older participants themselves using electronic food scales. Semi-quantitative recording (e.g., spoons, cups) is allowed, if accurate weighing is not possible. Information on recipes (ingredients and preparation) and on the types and brands of commercial food products is also required. Medication and dietary supplement use were excluded for the present analyses. Energy and nutrient intake are calculated using the continually updated in-house food composition database LEBTAB [18]. The composition of basic foods is based on the German food composition tables BLS 3.02. Energy and nutrient contents of commercial food products, i.e., canned foods, ready to eat meals or snacks, are estimated by recipe simulation using labeled ingredients and nutrient contents [18].

### Definitions of outcome variables

In the current analyses, total dairy and different types of dairy were examined as outcome variables (Table 1). Total Dairy (TD) was defined as all dairy products (Table 1) and calculated in g/1000 kcal Total Energy Intake (TEI) per day. In addition, dairy products were further subdivided into the following in part overlapping dairy types. In terms of nutrient content, we specified Low Fat Dairy (LFD) compared to high-fat dairy and High Sugar Dairy (HSD) compared to low sugar dairy (Table 1). According to fermentation status, we defined Fermented Dairy (FD) compared to non-fermented dairy. Finally, Liquid Dairy (LD) was defined as dairy consumed as a beverage versus food dairy that was eaten (Table 1). All types of dairy were calculated as weight percent of TD intake (w%TD).

**Table 1:** Food group classification of dairy products^a^, DONALD study

Classified food group	Foods
Total dairy (TD)	All dairy products (including dairy from cows and other mammals, such as goats or sheep), excluding cream cakes and ice cream, because they are consumed as sweets rather than to meet dairy requirements, and excluding butter
Liquid dairy (LD)	Fresh milk, not-fermented and fermented drinks (e.g., cacao, buttermilk, whey), liquid sour milk products (incl. squeeze sour milk), yogurt drink
Fermented dairy (FD)	*Fermented liquid and solid dairy*: fermented dairy drinks (buttermilk, whey), liquid sour milk products (incl. squeeze sour milk), yogurt drink, yogurt, firm sour milk products, fermented desserts, fresh cheese, quark, cream fraiche, *cheese* (soft cheese, sliced cheese, hard cheese, and processed cheese)
Low fat dairy^b^ (LFD)	Non-fermented and fermented beverage dairy, non-fermented solid dairy, fermented solid dairy (fresh cheese, quark) < 2% fat, fresh cheese, quark (< 9% fat), soft cheese, processed cheese (< 15% fat), semi-hard and hard cheese (< 18% fat)
High sugar dairy^c,d^ (HSD)	Added sugar > 7 g/100 g industrially sweetened dairy

^a^ Dairy products can occur in different groups

^b^ Classification based on https://www.lebensmittellexikon.de/f0000170.php

^c^ Including instant powders for milk (i.e., cocoa)

^d^ Defined by the 1st quartile (6.9 g) added sugar/100 g in the 965 sweetened products reported by the study sample

For simplicity, only the four dairy types LFD, HSD, FD and LD are presented below, since they correspond to each other anyway with their respective pairs of opposites with reversed signs.

### Assessment of potential confounding factors

The following variables were taken into consideration as potential confounding factors: sex (boy/girl), body weight status (normal weight, overweight, obesity, underweight), number of weekdays per 3-day record (1/2/3), maternal overweight status (normal weight, overweight, obesity), high maternal educational status (yes/no), and maternal employment (yes/no).

Weight and height of the participants were measured by study nurses according to standard procedures [16], dressed only in underwear and barefoot using an electronic scale (Seca 753E; Seca Weighing and Measuring System,  ±100 g) and a digital stadiometer (Harpenden, Crymych, UK, ±0.1 cm). Body mass index (BMI [kg/m^2^]) was calculated as the body weight (kg) divided by the square of the body height (m^2^). Overweight was determined corresponding to International Obesity Task Force’s (IOTF) BMI cutoff values for children and adolescents [19, 20]. Maternal overweight was defined as a BMI ≥ 25 to < 30 kg/m^2^, maternal obesity as a BMI > 30 kg/m^2^.

High maternal educational status (≥ 12 school years) and maternal employment are surveyed using a standardized questionnaire. Missing values were replaced by the respective median of the total sample (*n* = 127 for maternal overweight, *n* = 16 for maternal educational status).

### Statistical analysis

All statistical analyses were carried out using SAS^®^ procedures (version 9.20 and 9.40; Cary, NC, USA). The significance level was set at *p* < 0.05. Descriptive data are shown as median with their interquartile range or frequencies and percentages. We stratified the data for descriptive purpose according to the following age groups: 3.5 < 6.5 years (*n* = 8256), 6.5 < 10.5 years (*n* = 9588), 10.5 < 14.5 years (*n* = 7587) and 14.5 ≤ 18.5 years (*n* = 5568) (Table 2) and to the following time periods: 1985–1989, 1990–1994, 1995–1999, 2000–2004, 2005–2009, 2010–2014, 2015–2019) (Supplemental Table 1).

**Table 2:** Sample and dietary characteristics of *n* = 10,333 3-day-weighted dietary records of *n* = 1275 DONALD (boys *n* = 648, girls *n* = 627) participants aged 3.5–18.5 years, collected between 1985 and June 2019, stratified by sex and age groups, DONALD study

	Boys				Girls			
	3.5 < 6.5	6.5 < 10.5	10.5 < 14.5	14.5 ≤ 18.5	3.5 < 6.5	6.5 < 10.5	10.5 < 14.5	14.5 ≤ 18.5
*n*_participants_^a^	556	505	407	294	539	471	376	289
*n*_3-day-dietary-records_ (%)	1392 (26)	1644 (31)	1300 (25)	943 (18)	1360 (27)	1552 (31)	1229 (24)	913 (18)
Age (years)	5.0 (4.1; 6.0)	8.2 (7.2; 9.2)	12.2 (11.2; 13.2)	16.2 (15.2; 17.2)	5.0 (4.1; 6.0)	8.2 (7.3; 9.2)	12.2 (11.2; 13.2)	11.2 (15.2; 17.3)
Underreported records	34 (2.4)	65 (4.0)	169 (13.0)	215 (22.8)	36 (2.7)	30 (1.9)	104 (8.5)	195 (21.4)
Anthropometrics								
BMI	15.6 (14.9; 16.4)	15.6 (14.8; 16.7)	17.7 (16.1; 19.5)	20.4 (18.5; 22.9)	15.5 (14.8; 16.4)	15.5 (14.7; 16.9)	17.3 (16.0; 19.6)	20.5 (18.8; 22.7)
Normal weight	452 (81.3)	415 (82.2)	312 (76.7)	215 (73.1)	421 (78.1)	345 (73.3)	279 (74.2)	219 (75.8)
Overweight	32 (5.8)	38 (7.5)	55 (13.5)	49 (16.7)	55 (10.2)	59 (12.5)	52 (13.8)	33 (11.4)
Obesity	3 (0.5)	10 (2.0)	9 (2.1)	11 (3.7)	5 (0.9)	5 (1.1)	5 (1.3)	5 (1.7)
Underweight^b^	69 (12.4)	42 (8.3)	31 (7.6)	19 (6.5)	58 (10.8)	62 (13.2)	40 (10.6)	32 (11.1)
Maternal characteristics								
Normal weight	376 (68.7)	341 (68.9)	259 (64.8)	173 (60.1)	366 (69.2)	312 (67.1)	224 (60.2)	174 (60.8)
Overweight	127 (23.2)	115 (23.2)	107 (26.8)	85 (29.5)	123 (23.3)	121 (26.0)	113 (30.4)	82 (28.7)
Obesity	44 (8.0)	39 (7.9)	34 (8.5)	30 (10.4)	40 (7.6)	32 (6.9)	35 (9.4)	30 (10.5)
High educational status^c^	387 (69.6)	321 (63.7)	241 (59.4)	174 (59.2)	353 (65.6)	294 (62.4)	222 (59.4)	171 (59.4)
Employment	243 (43.7)	296 (58.6)	269 (66.1)	213 (72.5)	238 (44.2)	260 (55.2)	246 (65.4)	196 (67.8)
Dietary characteristics								
TEI (kcal/day)	1353 (1195; 1520)	1720 (1518; 1922)	2010 (1742; 2310)	2439 (2083; 2810)	1235 (1094; 1384)	1545 (1356; 1720)	1772 (1525; 2021)	1778 (1503; 2054)
TD (g/1000 kcal of TEI)	213 (142; 293)	191 (124; 275)	170 (100; 246)	151 (92; 223)	204 (132; 280)	174 (113; 247)	151 (93; 215)	143 (81; 215)
LFD (w%TD)	15.1 (1.5; 60.0)	16.8 (1.1; 60.5)	24.4 (1.4; 72.0)	28.4 (1.5; 75.8)	18.0 (1.6; 62.5)	21.1 (1.5; 62.9)	27.5 (1.3; 72.0)	34.0 (0.9; 72.8)
HSD (w%TD)	28.9 (12.2; 52.3)	24.1 (9.1; 46.5)	24.1 (8.5; 48.3)	18.9 (6.0; 41.5)	31.9 (14.7; 59.1)	29.6 (12.8; 53.9)	31.1 (13.0; 56.0)	27.6 (11.2; 50.8)
FD (w%TD)	20.3 (7.8; 41.3)	20.0 (7.9; 40.5)	23.6 (9.7; 46.4)	25.5 (10.2; 48.2)	22.0 (8.7; 41.1)	22.0 (9.0; 42.2)	27.8 (11.3; 51.7)	32.4 (16.1; 58.9)
LD (w%TD)	56.6 (29.4; 75.9)	52.2 (20.7; 73.1)	43.4 (0.0; 68.0)	40.4 (0.0; 67.2)	55.2 (27.3; 76.5)	51.2 (22.8; 72.3)	40.8 (0.0; 65.8)	34.6 (0.0; 63.4)

Values are *n* (%) or medians (25th, 75th percentile)

*TEI* total energy intake; *TD* total dairy; *LFD* low fat dairy; *HSD* high sugar dairy; *FD* fermented dairy; *LD* liquid dairy; *w%TD* intake weight as % of total dairy

^a^Due to repeated measurements per participant. One person can occur in more than one age group

^b^IOTF Criteria

^c^ > 12 years school education

Age and time trends in TD (g/1000 kcal TEI), LFD, HSD, FD, and LD (w%) were analyzed using polynomial regression models (PROC MIXED procedure in SAS) including both fixed and random statements.

A basic model was calculated for all variables, which included time in years (baseline was the first record included in the evaluation) and chronological age (years) as the most important fixed effects. Quadratic and cubic terms of age (age^2^, age^3^) and time (time^2^, time^3^) were considered as additional explanatory variables if they significantly predicted the respective outcome or improved the fit statistics [Akaike information criterion (AIC)] by more than two points [21]. There were no interactions between age and time (age × time) in all models.

A significant linear trend reflects a constant increase or decrease in the respective outcome variable over the years or with age. In contrast, quadratic and cubic trends indicate that the size of the trend changes over the study period or with age. A stratified analysis was conducted for significant sex interactions (sex × age, sex × time).

A repeated statement was considered so as to account for the lack of independence between repeated measures from the same person. Random effects were considered to allow variation between individuals and families with respect to the initial level (intercept) as well as linear, quadratic and cubic age trends of the respective outcome. The AIC was also used to select the covariance structure that best described the variances and covariances of the initial level, the linear and quadratic trend among persons, and the covariance structure that best describes the correlated nature of the repeated measurements. The variables remained in the final models if they had a significant and independent association with the outcome variable (*p* < 0.05), if the regression coefficients in the basic models were modified by ≥ 10% or if they led to an improvement in AIC of more than two points.

As single effect estimates of polynomial models cannot be interpreted, figures show the predicted trends for TD, LFD, HSD, FD, and LD resulting from the polynomial mixed-effects regression models over the course of the study period for different ages. Thus, the course of the curves illustrates the time trend and the vertical differences between the curves of the different ages indicate the age trend.

To avoid possible bias in the results, sensitivity analyses were run excluding records defined as underreported. Records were classified as underreported when the TEI was inadequate in relation to the estimated basal metabolic rate (BMR) according to age- and sex-specific equations of Schofield [22]. Pediatric cutoffs from Sichert-Hellert et al. [23] were used to identify underreported records. This calculation resulted in 848 (8.21%) underreported records (Table 2). Underreported records were not excluded from the main analyses, as this method only identifies underreported energy intake, but not selective underreporting of food groups [24]. Furthermore, participants with high-energy requirements (e.g., high physical activity), who may have been underreported, could not be identified [25]. Sensitivity analyses excluding the underreported datasets showed similar results in time and age trends.

## Results

The sample of the present evaluation includes all available complete dietary records (*n* = 10,033) of *n* = 1275 participants (*n* = 648, boys 50.82%) aged 3.5–18.5 years collected between August 1985 and June 2019. Per participant, a minimum of 1 (*n* = 144; 11.3%) to a maximum of 15 (*n* = 207;16.2%) dietary records [median (Q1; Q3): 8 (5; 12)] were available.

Sample characteristics are shown in Table 2. About half of the participants were female. The overweight status and maternal characteristics of the participants reflect the high socio-economic status of the DONALD study participants.

**Fig. 1:**
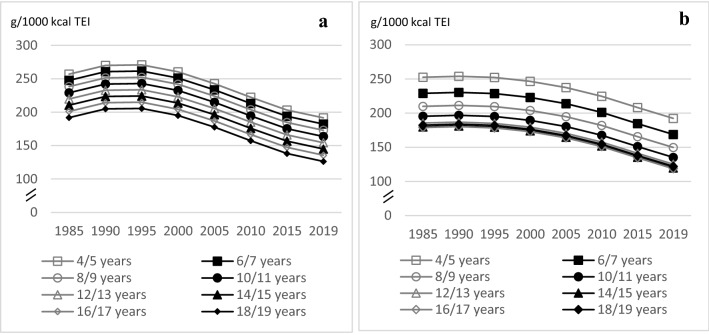
Age and time trends in TD g/1000 kcal TEI in boys (**a**) and in girls (**b**) of 10,333 3-day-weighted dietary records of 648 male and 627 female DONALD study participants (3.5–18.5 years) between 1985 and 2019, predicted by polynomial mixed-effects regression models (see Table 3)

**Fig. 2:**
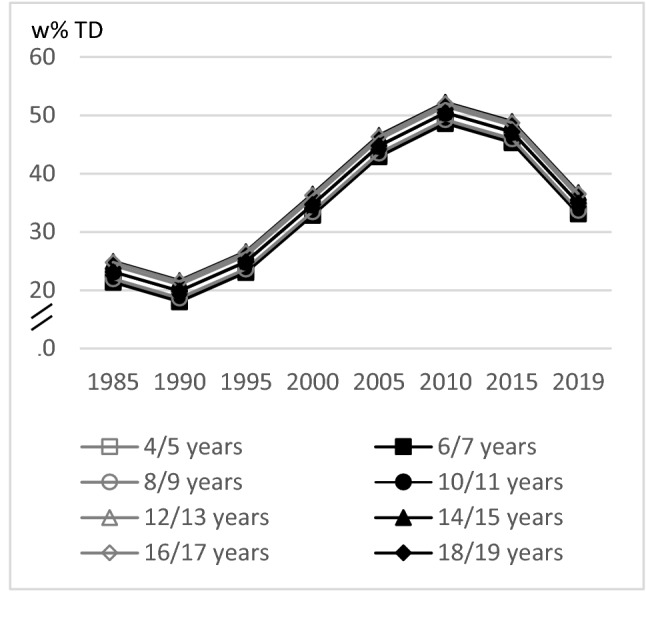
Age and time trends in LFD w%TD in boys and girls of 10,333 3-day-weighted dietary records of 648 male and 627 female DONALD study participants (3.5–18.5 years) between 1985 and 2019, predicted by polynomial mixed-effects regression models (see Table 3)

**Fig. 3:**
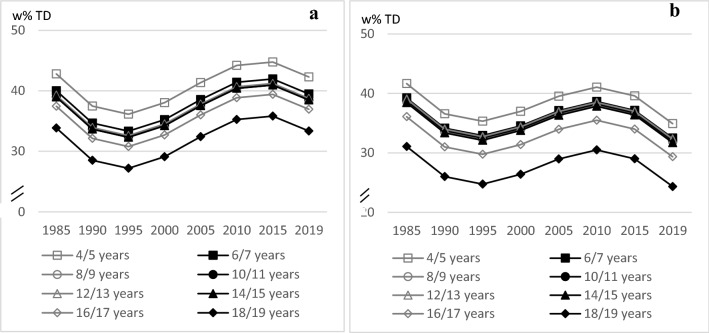
Age and time trends in HSD w% in boys (**a**) and in girls (**b**) of 10,333 3-day-weighted dietary records of 648 male and 627 female DONALD study participants (3.5–18.5 years) between 1985 and 2019, predicted by polynomial mixed-effects regression models (see Table 3)

**Fig. 4:**
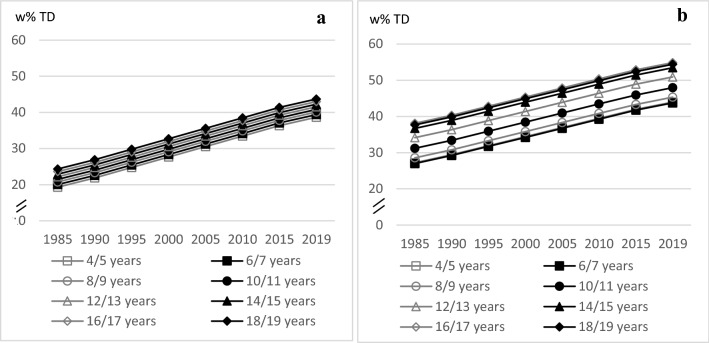
Age and time trends in FD w% in boys (**a**) and in girls (**b**) of 10,333 3-day-weighted dietary records of 648 male and 627 female DONALD study participants (3.5–18.5 years) between 1985 and 2019, predicted by polynomial mixed-effects regression models (see Table 3)

**Fig. 5:**
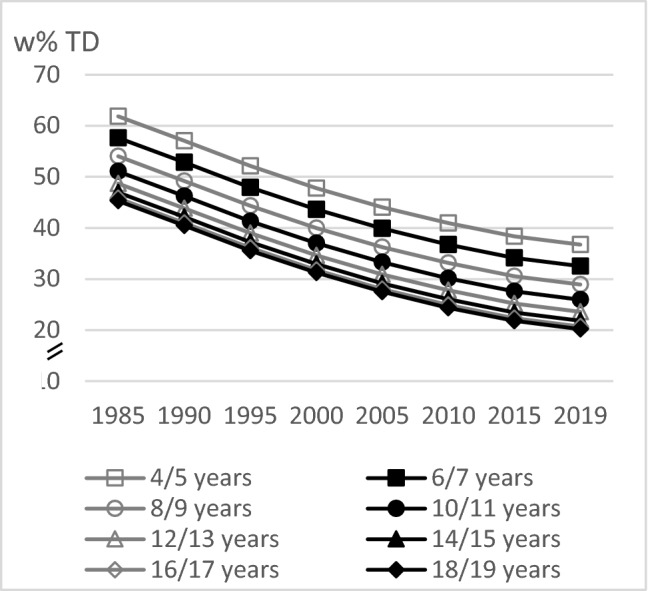
Age and time trends in LD w%TD in boys and girls of 10,333 3-day-weighted dietary records of 648 male and 627 female DONALD study participants (3.5–18.5 years) between 1985 and 2019, predicted by polynomial mixed-effects regression models (see Table 3)

Dietary characteristics stratified by age groups and sex are also displayed in Table 2. Intake of dairy and dairy types stratified by time periods and sex are presented in supplemental Table 1. The results of age and time trend analyses are presented in Table 3 and in Figs. 1–5.

**Table 3:** Age and time trends in dairy intake of *n* = 10,333 dietary records of *n* = 1275 DONALD participants, aged 3.5–18.5 years, collected between 1985 and 2019

	Age trend per year of age (3.5–18.5 years)^a^			Time trend per study year (1985–2019)^b^		
	Age	Age^2^	Age^3^	Time	Time^2^	Time^3^
	ß (*p*)	ß (*p*)	ß (*p*)	ß (*p*)	ß (*p*)	ß (*p*)
*Total dairy (g/1000 kcal TEI)*						
Boys^c^						
Unadjusted model	−4.5844 (< 0.0001)'			0.6491 (0.5021)'	−0.08839 (0.0006)'	
Adjusted model	−4.5746 **(< 0.0001)**'			0.3909 (0.6899)'	−0.08737 **(0.0007)**'	
Girls^d^						
Unadjusted model	−16.4255 (< 0.0001)'	0.5383 (< 0.0001)'		0.7199 (0.4599)'	−0.07368 (0.0041)'	
Adjusted model	−16.7772 **(< 0.0001)**'	0.5411 **(< 0.0001)'**		0.7893 (0.4087)'	−0.07306 **(0.0044)**'	
Low fat dairy (w%TD)^h^						
Unadjusted model	−3.1081 (0.0233)'	0.3424 (0.0114)'	−0.01094 (0.0085)'	−1.7281 (0.0002)'	0.2667 (< 0.0001)'	−0.00609 (< 0.0001)'
Adjusted model	−2.4395 (0.0773)'	0.3009 **(0.0267)**'	−0.00989 **(0.0177)**'	−1.8235 **(< 0.0001)**'	0.2706 **(< 0.0001)**'	−0.00615 **(< 0.0001)**'
*High sugar dairy (w %TD)*						
Boys^i^						
Unadjusted model	−5.9030 (0.0009)'	0.5367 (0.0022)'	−0.01660 (0.0020)'	−1.8524 (0.0007)'	0.1302 (0.0002)'	−0.00235 (0.0004)'
Adjusted model	−5.2977 **(0.0028)**'	0.5114 **(0.0035)**'	−0.01600 **(0.0029)**'	−1.7557 **(0.0013)**'	0.1346 **(0.0001)**'	−0.00247 **(0.0002)**'
Girls^j^						
Unadjusted model	−5.4697 (0.0040)'	0.5581 (0.0029)'	−0.01851 (0.0013)'	−1.6730 (0.0035)'	0.1324 (0.0004)'	−0.00263 (0.0002)'
Adjusted model	−5.3363 **(0.0049)**'	0.5517 **(0.0032)**'	−0.01834 **(0.0014)**'	−1.6804 **(0.0033)**'	0.1318 **(0.0004)**'	−0.00262 **(0.0002)**'
Fermented dairy (w%TD)						
Boys^f^						
Unadjusted model	0.2497 (0.0413)'			0.5855 (< 0.0001)'		
Adjusted model	0.3594 **(0.0192)**'			0.5800 **(< 0.0001)**'		
Girls^g^						
Unadjusted model	−4.7073 (0.0066)'	0.5148 (0.0028)'	−0.01486 (0.0050)'	0.5058 (< 0.0001)'		
Adjusted model	−3.9043 **(0.0248)**'	0.4955 **(0.0040)**'	−0.01517 **(0.0041)**'	0.5013 **(< 0.0001)**'		
Liquid dairy (w%TD)^e^						
Unadjusted model	−2.4665 (< 0.0001)'	0.06682 (0.0010)'		−1.1254 (< 0.0001)'	0.01157 (0.0353)'	
Adjusted model	−2.8791 **(< 0.0001)**'	0.07708 **(0.0002)**'		−1.1428 **(< 0.0001)**'	0.01170 (**0.0329**)'	

Significant *p*-values in the adjusted model are bolded

*w%TD* intake weight as % of TD; *TEI* total energy intake (kcal/day)

Age and time trends were tested using polynomial mixed-effect regression models

^a^ Age = linear age trend, age^2^ = quadratic age trend, age^3^ = cubic age trend

^b^ Time = linear time trend, time^2^ = quadratic time trend, time^3^ = cubic time trend

^c^ Model contains a random statement for the family level with an unstructured covariance structure and a random statement for the person level with an unstructured covariance structure adjusted for number of weekdays per record (1/2/3), high maternal educational status (yes/no), maternal overweight status (normal weight, overweight, obesity)

^d^ Model contains a random statement for the family level with an unstructured covariance structure and a random statement for the person level with a Huynh–Feldt covariance structure adjusted for number of weekdays per record (1/2/3)

^e^ Model contains a random statement for the family level with an unstructured covariance structure and a random statement for the person level with a no diagonal factor analytic covariance structure adjusted for TEI and for number of weekdays per record (1/2/3)

^f^ Model contains a random statement for the family level with an unstructured covariance structure and a random statement for the person level with an unstructured covariance structure adjusted for TEI

^g^ Model contains a random statement for the family level with a Huynh–Feldt covariance structure and a random statement for the person level with an unstructured covariance structure adjusted for TEI

^h^ Model contains a random statement for the family level with an unstructured covariance structure and a random statement for the person level with an unstructured covariance structure adjusted for TEI, overweight status (normal weight, overweight, obesity, and underweight), maternal overweight status (normal weight, overweight, obesity)

^i^ Model contains a random statement for the family level with an unstructured covariance structure and a random statement for the person level with an unstructured covariance structure adjusted for overweight status (normal weight, overweight, obesity, and underweight), TEI, high maternal educational status (yes/no)

^j^ Model contains a random statement for the family level with an unstructured covariance structure and a random statement for the person level with a no diagonal factor analytic covariance structure adjusted for number of weekdays per record (1/2/3)

^k^ Model contains a random statement for the family level with an unstructured covariance structure and a random statement for the person level with an unstructured covariance structure adjusted for number of weekdays per record (1/2/3) and overweight status (normal weight, overweight, obesity, and underweight)

^l^ Model contains a random statement for the family level with a no diagonal factor analytic covariance structure and a random statement for the person level with a no diagonal factor analytic covariance structure adjusted for number of weekdays per record (1/2/3) and high maternal educational status (yes/no)

^m^ Model contains a random statement for the family level with an unstructured covariance structure and a random statement for the person level with an unstructured covariance structure adjusted for number of weekdays per record (1/2/3)

### Total dairy

TD intake decreased with age in boys and girls (♂: linear trend *p* < 0.0001; ♀: linear and quadratic trend *p* < 0.0001), but for girls the intake is about the same among the 12–18-year-olds (Fig. 1). In terms of time trends, TD intake increased slightly for boys until 1995 and then decreased thereafter until the end of the analysis period. For girls, the intake of TD was at a similar level between 1985 and 1995 and decreased since 1995 (quadratic trend ♂: *p* = 0.0007 and ♀: *p* = 0.0044) (Fig. 1).

### Dairy types

LFD intake was almost at a comparable level among the age groups (quadratic trend *p* = 0.0267, cubic trend *p* = 0.0177). The time trend analysis of LFD showed a slightly decrease between 1985 and 1990, a sharply increase since 1990 and finally in a strong decrease since 2010 (linear, quadratic, cubic trend *p* < 0.0001) (Fig. 2).

The age trend analysis of HSD showed the highest HSD intake in the youngest and the lowest intake in the oldest age group in both, boys and girls (♂: linear trend *p* = 0.0028, quadratic trend *p* = 0.0035, cubic trend *p* = 0.0029; ♀: linear trend *p* = 0.0049, quadratic trend *p* = 0.0032, cubic trend *p* = 0.0014). Differences between the other age groups were negligible. In terms of time trends, HSD intake decreased between 1985 and 1995 in both sexes, increased in girls until 2010 and in boys until 2015 and decreased again afterwards in both sexes (♂: linear trend *p* = 0.0013, quadratic trend *p* = 0.0001, cubic trend *p* = 0.0002; ♀: linear trend *p* = 0.0033, quadratic trend *p* = 0.0004, cubic trend *p* = 0.0002) (Fig. 3).

FD intake did not differ much between the age groups for boys. For girls there was a slight age trend with the highest intake in the oldest and the lowest intake in the youngest age group (♂: linear trend *p* = 0.0192; ♀: linear trend *p* = 0.0248, quadratic trend *p* = 0.0040, cubic trend *p* = 0.0041). FD intake increased almost linear throughout the 30-year study period (♂ and ♀: linear trend *p* < 0.0001) (Fig. 4).

LD intake decreased slightly with age among the 16–18-year-olds, and it did not differ much (linear trend *p* < 0.0001, quadratic trend *p* = 0.0002). The time trend analyses in LD intake showed an almost linear decrease between 1985 and 2019 (linear trend *p* < 0.0001, quadratic trend *p* = 0.0329) (Fig. 5).

## Discussion

Based on a unique and comprehensive longitudinal database, the present study identified age and time trends in the intake of TD and as a first for its types (LFD, HSD, FD and LD) among children and adolescents in Germany between 1985 and 2019.

TD intake (g/1000 kcal TEI) decreased with age and time. The observed time trend is in line with the findings of the review by Dror et al. showing a decline of dairy consumption among children and adolescents in developed countries over the past three decades [26]. In addition, the current results of the European IDEFICS (Identification and prevention of Dietary- and lifestyle-induced health Effects In Children and infantS) study suggests a decline of dairy intake between 2007/2008 and 2013/2014, most notably at snack bar events [27].

As meta-analyses with children [1, 2] showed health benefits of dairy intake, the observed decrease in TD appears to be critical. However, it is not yet clear how much dairy should be consumed by children and adolescents. While some European countries, such as the Nordic countries and Spain, do not provide quantitative but only qualitative recommendations for dairy intake, most countries generally recommend two to three servings daily [28]. The German dietary guidelines recommend a daily intake of about 260 g/1000 kcal corresponding to the three servings of dairy [29]. Median intake among DONALD participants in the current investigation was lower, in particular in older study participants. However, Canada’s Food Guide as well as the newly developed EAT Lancet diet focus on plant protein, leaving a less prominent role for dairy products despite the importance of this food group on health [30, 31]. However, evidence stems mainly from adult populations. In addition, recent research suggests that the total dairy approach may not be meaningful because different types of dairy may affect health in different ways [38, 52, 53]. Hence, further studies are necessary about the impact of dairy intake as part of overall dietary pattern on health status especially in children and adolescents.

Among the dairy types, particularly pronounced age trends were observed for LD and HSD w%TD. The decreasing age trend for LD w%TD with the highest intake rates among the youngest possibly could be attributed to the ongoing transition from the liquid infant formula to milk. The observed age trend in HSD w%TD also with the highest intake rates among the youngest may be attributed to a higher sweet preference during childhood than late adolescence [32, 33].

In terms of time, our analysis showed the strongest trends for LD and FD w%TD, with a decreasing LD and an increasing FD intake. In the above mentioned trend analysis of DONALD participants [14] which covered the period from 1986 to 2001, the reduced intake of LD was compensated by increased intake of solid dairy (i.e., to be eaten with spoons) whereas the overall dairy intake remained stable. In contrast, the present analysis showed that in the past decade, the shift from LD to solid dairy was associated with a general downward trend of TD intake. To our knowledge, there are no other trend analyses of LD or solid dairy, to which our results could be compared. Only in Canada, a descriptive comparison showed a reduction of the consumption of fluid milk in adults over the past two decades with a rise in the consumption of solid dairy foods, such as cheese and yogurt [30]. As in the case of sugar-sweetened beverages, liquid dairy are supposed to cause less satiation than solid dairy [11]. Overweight/obese children from the Avon Longitudinal Study consuming flavored milk had less favorable changes in body composition over time [34]. Whether this is due more to sugar content or consistency remains to be investigated. In contrast, FD w%TD increased significantly over time. At the end of the observation period, fermented products even accounted for almost a third of TD intake. To our knowledge, no other studies analyzed age and time trends in FD intake. However, it should be noted, that various meta-analyses have indicated a benefit of consuming FD [4, 9, 33, 35, 36]. Hence, the observed increase in FD w%TD among children and adolescents should be considered as a positive development.

We observed further time trends in LFD and HSD w%TD. There was an initially strong increase of LFD w%TD, similar to the aforementioned former evaluation of DONALD study data [14] from 1986 until 2001. Our analyses showed that this trend continued until 2010 and sharply declined afterwards. While in 2003 the majority of food-based dietary guidelines in Europe recommended consumption of two to three servings of low-fat dairy products per day [37], these recommendations have been relativized in terms of fat content in some countries, such as Germany and Austria, as recent studies provide hints of potential health benefits from consumption of full-fat dairy products [38–42]. Currently, the effect of fat content in consumed dairy products on body weight in children is being studied in the Cow’s Milk Fat Obesity pRevention Trial (CoMFORT) [43].

Similar to our time trend in HSD intake, a previous investigation of the DONALD study showed that free sugar intake from dairy increased slightly between 1985 and 2010 and decreased thereafter [7]. In contrast to these findings, HSD intake also decreased between 1985 and 1995. HSD intake accounted for about 19–32% of total dairy intake in the time period 2015–2019. There is a potential to reduce the amount of added sugar in dairy products by reformulation [44], in particular as natural lactose content results in a sweet taste of unfermented dairy. As the free sugar intake in Germany exceeded the limit of 10% of energy intake, a further reduction would be desirable [7, 45]. In addition, consumers should become aware that sweetened dairy products contribute to a relevant amount to free sugar intake [46] and encouraged to select unsweetened products. It should be noted that only industrially sweetened dairy has been taken into account in our study, whereas sugar or other sweeteners added in the household was not considered. Therefore, the overall contribution of sugar-sweetened dairy intake might be higher.

Some strengths and limitations of the DONALD study have to be discussed: The major strength of this study is the longitudinal design, which allows time and age trend analyses over a period of three decades in a large sample size using a large number of 3-day weighted dietary records. The continuously updated in-house nutrient database LEBTAB allows a consideration of different dairy types and their ingredients [18]. A limitation of the present study is the overrepresentation of families with a high socio-economic background in the collective of the DONALD study, which limits the generalizability of our results [17]. However, in the age group 14–18, our intake data are comparable to the results of the German National Food Consumption Study (Nationale Verzehrsstudie, NVS II) [47].

In addition, we cannot rule out the possibility of underreporting. However, our sensitivity analyses did not support the notion of bias from general underreporting, and the results for age and time trends were similar when we excluded underreported records.

## Conclusion

Dairy consumption has been recommended as part of a healthy diet [48], as meta-analyses showed a significant inverse association of total dairy consumption with diabetes [49], Cancer [50] and Hypertension [51]. However, recent research suggests that the total dairy approach may be too simplistic, as dairy types may affect health in different ways [38, 52, 53]. In this article, we were able to show for the first time changes in the consumption patterns of dairy in children and adolescents over the past three decades, demonstrating for example, a switch from liquid to solid dairy with a simultaneous increase in fermented dairy. Further evaluations will investigate the health significance of these consumption patterns.

## Supplementary Information

Below is the link to the electronic supplementary material.

Supplementary file1 (DOCX 242 KB)
