# Supplementary material for: Age and time trends of dairy intake among children and adolescents of the DONALD study
Source: Eur J Nutr. 2021 Apr 21;60(7):3861–72. doi: 10.1007/s00394-021-02555-7 (PMC8437911; doi:10.1007/s00394-021-02555-7)
Supplement: Supplementary file 1 — Supplementary file1 (DOCX 242 KB) [file 394_2021_2555_MOESM1_ESM.docx]

**Supplemental Table 1:** Dietary characteristics from n=10,333 3-day weighted dietary records of n=1,275 DONALD participants aged 3.5 to18.5 years, collected between 1985 and 2019, stratified by time and sex, DONALD Study

| \|  \| boys \| \| \| \| \| \| \| \| --- \| --- \| --- \| --- \| --- \| --- \| --- \| --- \| \| time \| 1985-1989 \| 1990-1994 \| 1995-1999 \| 2000-2004 \| 2005-2009 \| 2010-2014 \| 2015-2019 \| \| TEI (kcal/day) \| 1767(1426; 2114) \| 1633(1370; 1924) \| 1744 (1458; 2096) \| 1788 (1481; 2242) \| 1829 (1467; 2283) \| 1795 (1466; 2170) \| 1744 (1445; 2148) \| \| EI Total-Dairy (kcal/day) \| 327 (237; 450) \| 321 (225; 443) \| 342 (232; 459) \| 315 (215; 445) \| 299 (208; 427) \| 268 (175; 389) \| 254 (169; 366) \| \| TD (g/1000kcal) \| 227 (157; 300) \| 224 (149; 312) \| 209 (146; 284) \| 193 (123; 266) \| 174 (110; 254) \| 151 (96; 228) \| 143 (83; 209) \| \| LFD (w%) \| 5 (0.8; 16.4) \| 4.8 (0.3; 21.4) \| 6.0 (0.0; 34.5) \| 19.3 (1.1; 67) \| 49.4 (10.8; 78.8) \| 51.4 (5.5; 80.2) \| 36.9 (2.4; 72.7) \| \| HSD (w%) \| 24.1 (9.5; 53.1) \| 20.0 (8.4; 42.7) \| 20.9 (8.1; 41.8) \| 23.8 (8.2; 47.7) \| 26.8 (11.0; 49.8) \| 26.5 (9.8; 51.3) \| 26.4 (9.4; 53.2) \| \| FD (w%) \| 12.9 (3.5; 25.9) \| 14.5 (5.6; 28.9) \| 17.5 (6.7; 33.1) \| 22.2 (9.9; 45.0) \| 26.7 (11.2; 51.6) \| 31.1 (12.0; 53.3) \| 29.8 (13.8; 53.8) \| \| LD (w%) \| 67.7 (43.2; 84.1) \| 61.5 (34.8; 80.0) \| 52.7 (21.2; 73.5) \| 46.7 (13.1; 69.9) \| 48.2 (17.2; 69.1) \| 42.2 (0.0; 66.6) \| 34.1 (0.0; 62.6) \| |
| --- | --- | --- | --- | --- | --- | --- | --- | --- | --- | --- | --- | --- | --- | --- | --- | --- | --- | --- | --- | --- | --- | --- | --- | --- | --- | --- | --- | --- | --- | --- | --- | --- | --- | --- | --- | --- | --- | --- | --- | --- | --- | --- | --- | --- | --- | --- | --- | --- | --- | --- | --- | --- | --- | --- | --- | --- | --- | --- | --- | --- | --- | --- | --- | --- | --- | --- | --- | --- | --- | --- | --- | --- |
| \|  \| girls \| \| \| \| \| \| \| \| --- \| --- \| --- \| --- \| --- \| --- \| --- \| --- \| \| time \| 1985-1989 \| 1990-1994 \| 1995-1999 \| 2000-2004 \| 2005-2009 \| 2010-2014 \| 2015-2019 \| \| TEI (kcal/day) \| 1498 (1249; 1753) \| 1438 (1227; 1704) \| 1512(1255; 1782) \| 1544 (1295; 1850) \| 1563 (1328; 1862) \| 1565 (1317; 1840) \| 1574 (1320; 1876) \| \| EI Total-Dairy (kcal/day) \| 257 (172; 364) \| 281 (202; 387) \| 269 (180; 380) \| 249 (168; 353) \| 245 (168; 336) \| 234 (156; 326) \| 214 (138; 309) \| \| TD (g/1000kcal) \| 201 (18; 77) \| 212 (142; 289) \| 192 (125; 263) \| 171(107; 238) \| 160 (107; 233) \| 146 (87; 222) \| 129 (80; 186) \| \| LFD (w%) \| 6.4 (0.6; 23.5) \| 7.7 (0.9; 29.7) \| 10.1 (0.6; 43.4) \| 22.9 (1.1; 68.2) \| 52.3 (9.3; 81.4) \| 48.7 (7.3; 77.3) \| 37.2 (1.1; 70.2) \| \| HSD (w%) \| 29.5 (10.2; 63.7) \| 28.1 (11.9; 52.8) \| 27.0 (12.0; 47.8) \| 30.3 (12. 9; 53.7) \| 32.1 (13.7; 59.6) \| 33.3 (16.3; 59.1) \| 32.3 (12.4; 57.1) \| \| FD (w%) \| 18.0 (5.6; 34.5) \| 19.8 (7.9; 35.1) \| 20.4 (8.4; 39.1) \| 25.1 (9.6; 45.7) \| 29.4 (12.7; 52.2) \| 31.9 (15.0; 57.5) \| 32.8 (14.4; 60.9) \| \| LD (w%) \| 64.0 (31.5; 79.8) \| 57.1 (33.12; 74.82) \| 50.1 (23.9; 72.5) \| 45.2 (6.5; 69.2) \| 45.5 (7.9; 67.8) \| 40.4 (0.00; 68.36) \| 35.1 (0.0; 62.1) \| |

Values are medians (25^th^, 75^th^ percentile)

TEI = Total Energy Intake,

EI = Energy Intake,

TD = Total Dairy,

LFD = Low Fat Dairy,

HSD = High Sugar Dairy,

FD = Fermented Dairy, L

D = Liquid Dairy,

w%TD = weight percent of Total Dairy
